# Global, regional, and national burden of early-onset colorectal cancer attributable to high BMI (1990–2021)

**DOI:** 10.3389/fmed.2025.1631392

**Published:** 2025-09-17

**Authors:** Yin Zhang, Fuzhou Han, Jun Qu

**Affiliations:** Department of General Surgery, Aerospace Center Hospital, Beijing, China

**Keywords:** global burden of disease, early-onset colorectal cancer, high BMI, disability-adjusted life years, colorectal cancer

## Abstract

**Objective:**

This study aimed to assess the global, regional, and national burden of early-onset colorectal cancer (EO-CRC) attributable to high body mass index (BMI) from 1990 to 2021. By examining demographic, geographic, and sex-specific disparities, the goal was to elucidate trends and inform targeted prevention strategies.

**Methods:**

We utilized data from the Global Burden of Disease (GBD) 2021 study, applying population-attributable fraction modeling to estimate disability-adjusted life years (DALYs) linked to high BMI (≥25 kg/m^2^). Analyses were stratified by sex, age, socio-demographic index (SDI), and geography. Temporal trends were assessed using Joinpoint regression, and future projections were estimated via Bayesian Age-Period-Cohort (BAPC) modeling. Decomposition and frontier analyses were conducted to identify key drivers of change and benchmark national performance.

**Results:**

Globally, age-standardized DALY rates (ASDRs) for EO-CRC attributable to high BMI increased from 8.07 per 100,000 in 1990 to 10.49 in 2021. The burden was consistently higher and increased more rapidly among males. While high SDI countries experienced stabilization or even decline in rates after 2017, sharp increases were observed in low-middle and low SDI countries. Population growth and epidemiological transitions were the primary contributors to DALY increases. Projections suggest further growth in burden, especially among males and in lower SDI regions.

**Conclusion:**

High BMI plays a substantial role in the rising global burden of EO-CRC, with pronounced disparities across regions and sexes. The shifting burden toward low and middle SDI countries, along with accelerating trends among men, highlights the urgent need for sex- and region-specific obesity prevention and early screening strategies to mitigate the growing public health impact of EO-CRC.

## 1 Introduction

Colorectal cancer (CRC) is among the most prevalent malignancies worldwide, currently ranked as the third most commonly diagnosed cancer and the second leading cause of cancer-related death globally (1). Early-onset colorectal cancer (EO-CRC), defined as CRC diagnosed before 50 years of age, has emerged as a growing public health concern (2). There is mounting evidence that EO-CRC incidence has been increasing in many parts of the world over the past few decades (3–5). Therefore, identifying the key risk factors for EO-CRC and developing targeted preventive strategies may represent one of the most effective approaches to reducing the risk of EO-CRC.

Among the hypothesized drivers of the global surge in EO-CRC, high body mass index (BMI)—defined as BMI ≥25 kg/m^2^—has attracted particular attention as a modifiable risk factor. High BMI is a well-established contributor to colorectal carcinogenesis in the general population (6). A large meta-analysis has estimated that CRC risk increases by approximately 7% for each 2 kg/m^2^ increment in adult BMI, and in 2019 roughly 8%−10% of all global CRC disability-adjusted life years (DALYs) were attributable to high BMI (7, 8). A substantial body of epidemiological evidence demonstrates a strong, dose-dependent relationship between elevated BMI and CRC risk, with some studies indicating a more pronounced association in men (6, 9, 10). Unlike non-modifiable risk factors (e.g., genetic predisposition), high BMI represents a preventable and reversible exposure, making it a critical target for public health intervention if its contribution to EO-CRC can be clearly demonstrated.

Despite growing recognition of EO-CRC and its potential link to rising obesity, most epidemiological studies have been confined to individual countries or regions, and comprehensive global analyses of EO-CRC and its risk factors remain scarce. This study provides a timely and comprehensive assessment of the global burden and temporal trends of EO-CRC attributable to high BMI, with a detailed analysis of sex- and region-specific disparities, based on data from the Global Burden of Disease (GBD) 2021 study. Understanding these patterns is crucial for informing targeted prevention and screening strategies.

## 2 Method

### 2.1 Study design and data sources

This study utilized data from the GBD 2021 study to assess the burden of EO-CRC attributable to high BMI from 1990 to 2021. DALYs for a disease or health condition are the sum of years of life lost due to premature mortality (YLLs) and years of healthy life lost due to disability (YLDs) due to prevalent cases of the disease or health condition in a population. Considering the dual nature of EO-CRC in contributing to both years of life lost and years lived with disability, DALYs provide a comprehensive measure of its burden and were therefore selected as the primary outcome metric. To facilitate comparisons across different regions and time periods, we used age-standardized DALY rates (ASDRs) in this study. ASDRs were derived by the direct method using the world standard population, multiplying each age-specific rate by the corresponding standard-population weight and dividing by the sum of weights. This method helps eliminate the influence of demographic differences in age distribution across regions or over time. This approach is a well-established methodology in GBD studies and is essential for revealing the genuine temporal trends in disease burden, rather than fluctuations caused by changes in population structure (11). The equation of ASDR is as following:


$$\begin{array}{l} \text{ASDR}=\frac{\sum_{i=1}^{N}DALY_{i}w_{i}}{\sum_{i=1}^{N}w_{i}} \end{array}$$

*DALY*_*i*_ is age-specific rate in age group *i, w*_*i*_ is the standard-population weight for the same age group; and *N* is the total number of age groups.

The GBD study synthesizes mortality, morbidity, and DALY's data for 204 countries and territories across 21 regions. Data sources include national vital registration systems, cancer registries, health surveys, hospital discharge records, and verbal autopsy data. The focus of the study was on EO-CRC, defined as CRC occurring in individuals aged under 50. The study specifically examined the disease burden resulting from high BMI, which is a significant risk factor for CRC. According to World Health Organization (WHO) criteria, individuals with a BMI ≥25 kg/m^2^ are classified as overweight or obese, and this classification was used to assess the contribution of high BMI to EO-CRC. The population attributable fraction (PAF) methodology was used to estimate the contribution of high BMI to EO-CRC burden. In the GBD 2021 framework, the disease burden attributable to high BMI is estimated based on the comparative risk assessment method, which integrates the population distribution of BMI, the relative risks of EO-CRC across BMI categories, and the theoretical minimum risk exposure level (TMREL)-typically defined as 20–25 kg/m^2^. The resulting PAF reflects the proportion of EO-CRC burden that could theoretically be avoided if the population BMI distribution were entirely shifted to the TMREL range. The data was extracted using the Global Health Data Exchange (GHDx) tool. In the GHDx interface, the screening criteria and strategies are as follows: (1) risk factor: high-BMI (major factor), high fasting plasma glucose, low physical activity, diet high in processed meat, diet high in red meat, diet low in calcium, diet low in fiber, diet low in milk, diet low in whole grains, high alcohol use, and high smoking; (2) age groups are 20–24, 25–29, 30–34, 35–39, 40–44 and 45–49 years; (3) location: global, 5 tier SDI regions and 21 GBD regions; (4) year: 1990 to 2021; (5) metric: number and rate; (6) cause: colon and rectum cancer; (7) sex: both, male and female. The analyses were performed using Joinpoint software (version 5.3.0), R (version 4.4.2) and JD_GBDR (V2.37, Jingding Medical Technology Co., Ltd.).

### 2.2 Trend analysis using Joinpoint regression

To analyze temporal trends, Joinpoint regression analysis was employed using Joinpoint software (version 5.3.0). The Joinpoint regression model is employed to analyze temporal patterns in disease distribution by establishing segmented regression based on time series data. Utilizing the grid search method (GSM), the model identifies significant inflection points where trends change. These Joinpoints divide the study period into distinct intervals, within which separate trend fitting and optimization are conducted. This approach enables a more refined evaluation of disease-specific temporal variation across different time segments. Furthermore, the model quantifies the annual percent change (APC) in disease rates within each interval, making it a widely used tool in epidemiological research to assess the impacts of policy interventions or environmental changes on disease trends (12). This method detects significant inflection points in the trend, where the direction or magnitude of the trend changes. APC and average annual percent change (AAPC) were calculated to quantify the rate of change in ASDRs over time. This analysis was performed globally and stratified by sex and socio-demographic index (SDI) quintile, allowing us to understand how the burden of EO-CRC attributable to high BMI has changed over time in different regions and for different subgroups.

### 2.3 Decomposition analysis

Decomposition analysis was used to evaluate the relative contributions of three factors—population growth, epidemiological change, and aging—to the increase in DALYs from 1990 to 2021. This method decomposes the total change in DALYs into the contributions of each factor, providing insights into the underlying drivers of the observed trends. The sum of contributions from all factors equals 100%, with positive contributions (greater than 0) represented by rightward bars and negative contributions (less than 0) represented by leftward bars in the bar chart. Black dots indicate the net change in DALYs for each stratum (13).

### 2.4 Frontier analysis

Frontier analysis was performed to evaluate the disease burden of EO-CRC attributable to high BMI across countries with varying SDI levels. This analysis was employed to assess the efficiency gap in disease control, health resource utilization, and public health interventions across regions, defined as the deviation from the theoretical optimal level (efficiency frontier). This approach enables the identification of countries or regions with room for improvement and serves as a benchmark for guiding policy optimization. For countries experiencing a worsening disease burden, these findings call for urgent attention and the implementation of targeted preventive strategies (14).

In the context of this study, the frontier refers to the best-performing countries in terms of reducing the burden of EO-CRC attributable to high BMI, as indicated by the lowest ASDRs. The countries located on or near the frontier represent those that have successfully reduced their disease burden or maintained it at low levels despite increasing BMI. Conversely, countries that are further away from the frontier may be experiencing higher growth in disease burden, signaling less effective control over the risk factor. Net-change analysis was conducted to quantify the change in DALYs for each country from 1990 to 2021. This involved examining the direction and magnitude of change in DALYs over the period, stratified by sex. By plotting these changes, we could identify countries that had improved health outcomes and those that had worsened.

### 2.5 Bayesian age-period-cohort (BAPC) modeling

BAPC modeling was performed to project the future trends in the burden of EO-CRC attributable to high BMI. This advanced statistical approach allows for a comprehensive analysis of the disease burden over time, accounting for three key components: age, period, and cohort. These components are essential for understanding how the disease burden changes across different generations and over time due to various socio-economic, environmental, and demographic factors. The BAPC framework is particularly appropriate for capturing the temporal dynamics of disease burden across diverse demographic strata. Its Bayesian nature enables the incorporation of prior information and facilitates the estimation of credible intervals, which improves both the robustness of the findings and their practical applicability in health policy planning (15). The projections produced by the BAPC model extend to the year 2040. These projections are based on current trends in age-specific rates of EO-CRC attributable to high BMI and are adjusted for uncertainty using 95% credible intervals.

## 3 Results

### 3.1 Relative contribution of high BMI to the increase in the burden of EO-CRC

**Figure 1:**
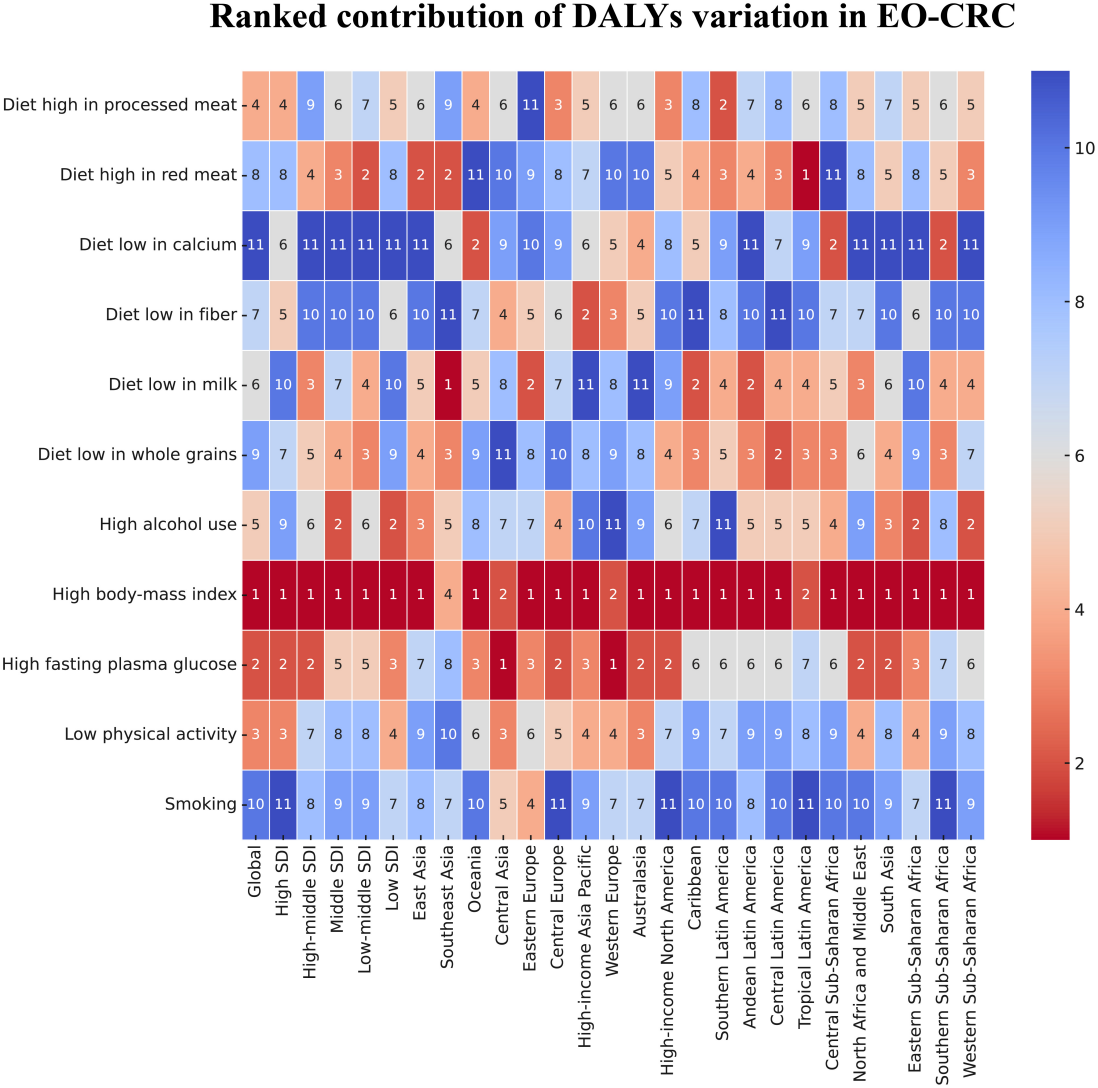
Ranked contribution of DALY variation in EO-CRC by 11 risk factors (1990–2021). This heatmap displays the rank of contribution (1 = largest, 11 = smallest) of each risk factor to the variation in DALYs for early-onset colorectal cancer (EO-CRC) across global, 5 SDI and 21 GBD regions. SDI, Socio-demographic Index; DALY, disability-adjusted life-year; EO-CRC, early-onset colorectal cancer.

Between 1990 and 2021, high BMI consistently ranked as the leading modifiable risk factor contributing to the DALYs rise in EO-CRC across nearly all global regions (Figure 1). Specifically, high BMI was the most significant factor among 11 modifiable exposures in 24 of the 27 analyzed locations, encompassing the global aggregate and all five SDI quintiles. For both men and women, high BMI ranked first in most regions, though there were regional differences (Supplementary Figures S1A, B). The largest increase in BMI-attributable EO-CRC cases for men occurred in East Asia (13.86%), whereas the increase for women in this region was only 4.23%. The greatest increase for women was seen in Tropical Latin America (9.80%), while the increase for men in this region was slightly lower at 9.66%.

### 3.2 The burden of EO-CRC attributable to high BMI at global, regional, and national levels

We further standardized the DALYs by age to explore the distribution of DALYs and estimated annual percentage change (EAPC) for EO-CRC attributable to high BMI across different SDI regions, 21 GBD regions, and 204 countries. Table 1 summarizes the age-standardized DALYs rates (ASDR) for EO-CRC attributable to high body-mass index in 1990 and 2021, along with the EAPC over the past three decades, disaggregated by sex, SDI quintile, and the 21 GBD regions. At the global level, the ASDR rose from 8.07 per 100,000 population (95% UI: 3.21–13.04) in 1990 to 10.49 (95% UI: 4.42–16.56) in 2021, representing a 30% relative increase, with a corresponding EAPC of 0.76% per year (95% CI: 0.69–0.82). The burden was consistently higher in men from 1990 to 2021 (from 8.22 to 11.80 per 100,000; EAPC: 1.12%, 95% CI: 1.06–1.18) compared to women (from 7.91 to 9.16; EAPC: 0.33%, 95% CI: 0.24–0.41). A clear SDI gradient was observed. High SDI locations experienced the greatest absolute burden in 2021 (15.22 per 100,000, 95% UI: 6.65–23.63), but showed only modest growth (EAPC: 0.53%, 95% CI: 0.48–0.59). In contrast, middle and low-middle SDI locations saw the most rapid increases (EAPC: 1.87% and 2.38%, respectively), with the latter doubling its ASDR from 3.04 to 6.15 per 100,000.

**Table 1:** The ASDR of prevalence of EO-CRC attributable to high BMI in 1990 and 2021 by SDI quintiles and by GBD regions, with EAPC from 1990 to 2021.

**Location**	**Both-ASDR (95% UI)**		**EAPC (95% CI)**	**Female-ASDR (95% UI)**		**EAPC (95% CI)**	**Male-ASDR (95%UI)**		**EAPC (95% CI)**
	**1990**	**2021**		**1990**	**2021**		**1990**	**2021**	
Global	8.07 (3.21, 13.04)	10.49 (4.42, 16.56)	0.76 (0.69, 0.82)	7.91 (3.17, 12.78)	9.16 (3.89, 14.40)	0.33 (0.24, 0.41)	8.22 (3.21, 13.41)	11.80 (5.03, 18.90)	1.12 (1.06, 1.18)
High SDI	13.07 (5.45, 20.94)	15.22 (6.65, 23.63)	0.53 (0.48, 0.59)	11.35 (4.70, 18.02)	12.74 (5.56, 19.67)	0.44 (0.39, 0.49)	14.76 (6.10, 23.87)	17.58 (7.59, 27.66)	0.58 (0.53, 0.64)
High-middle SDI	12.21 (4.88, 19.84)	14.83 (6.24, 24.02)	0.40 (0.25, 0.54)	11.90 (4.82, 19.40)	11.26 (4.77, 18.18)	−0.52 (−0.67, −0.36)	12.52 (4.86, 20.38)	18.25 (7.55, 30.54)	1.09 (0.95, 1.23)
Middle SDI	6.14 (2.25, 10.11)	11.01 (4.67, 17.47)	1.87 (1.79, 1.95)	6.20 (2.36, 10.19)	9.28 (4.04, 14.79)	1.19 (1.08, 1.30)	6.09 (2.12, 10.00)	12.71 (5.45, 20.64)	2.44 (2.38, 2.51)
Low-middle SDI	3.04 (1.14, 4.93)	6.15 (2.54, 9.83)	2.38 (2.31, 2.46)	4.02 (1.55, 6.61)	7.18 (3.00, 11.45)	1.97 (1.90, 2.04)	2.11 (0.74, 3.52)	5.11 (2.04, 8.31)	2.99 (2.89, 3.09)
Low SDI	2.36 (0.84, 4.03)	3.88 (1.55, 6.22)	1.58 (1.44, 1.71)	3.14 (1.11, 5.53)	4.49 (1.77, 7.37)	1.12 (0.99, 1.25)	1.59 (0.52, 2.78)	3.27 (1.22, 5.41)	2.31 (2.19, 2.42)
East Asia	7.22 (2.36, 12.26)	13.95 (5.68, 23.63)	2.04 (1.78, 2.29)	6.04 (1.93, 10.64)	7.98 (3.10, 14.21)	0.58 (0.32, 0.83)	8.30 (2.60, 14.45)	19.60 (7.82, 34.00)	2.83 (2.59, 3.07)
Southeast Asia	4.58 (1.68, 7.56)	9.35 (3.77, 15.30)	2.29 (2.11, 2.47)	5.15 (1.83, 8.95)	9.27 (3.81, 15.47)	1.95 (1.78, 2.13)	4.00 (1.39, 6.64)	9.42 (3.52, 15.72)	2.68 (2.48, 2.87)
Oceania	7.11 (2.87, 12.17)	7.62 (3.14, 12.87)	0.18 (0.12, 0.24)	7.85 (3.06, 14.00)	8.20 (3.34, 14.22)	0.10 (0.02, 0.17)	6.42 (2.55, 10.96)	7.06 (2.93, 11.76)	0.27 (0.21, 0.32)
Central Asia	13.49 (5.52, 21.93)	9.90 (4.05, 16.21)	−1.00 (−1.15, −0.85)	13.36 (5.54, 21.57)	9.38 (3.85, 15.34)	−1.18 (−1.28, −1.08)	13.61 (5.39, 22.45)	10.42 (4.25, 17.27)	−0.83 (−1.06, −0.60)
Central Europe	17.86 (7.53, 29.13)	16.32 (7.06, 26.54)	−0.39 (−0.54, −0.23)	14.47 (6.08, 23.57)	12.55 (5.48, 20.63)	−0.54 (−0.71, −0.38)	21.26 (8.87, 34.79)	19.97 (8.45, 32.29)	−0.31 (−0.47, −0.15)
Eastern Europe	17.10 (7.15, 27.00)	16.96 (7.08, 27.03)	−0.33 (−0.49, −0.18)	18.99 (8.16, 30.21)	16.30 (6.74, 26.27)	−0.83 (−0.99, −0.67)	15.07 (6.19, 23.99)	17.65 (7.21, 28.77)	0.24 (0.09, 0.40)
High-income Asia Pacific	7.09 (2.57, 11.76)	6.45 (2.51, 10.36)	−0.41 (−0.50, −0.31)	6.13 (2.18, 10.25)	5.12 (1.95, 8.46)	−0.69 (−0.88, −0.50)	8.02 (2.91, 13.29)	7.71 (3.00, 12.55)	−0.23 (−0.30, −0.16)
Australasia	17.17 (6.94, 27.91)	17.37 (7.59, 27.53)	0.08 (−0.12, 0.27)	16.66 (6.72, 27.50)	15.66 (6.83, 25.01)	−0.16 (−0.32, 0.01)	17.67 (7.03, 29.06)	19.14 (8.26, 30.83)	0.30 (0.07, 0.53)
Western Europe	12.43 (5.08, 20.45)	10.40 (4.48, 16.96)	−0.58 (−0.65, −0.51)	10.92 (4.45, 17.85)	9.18 (3.89, 14.86)	−0.54 (−0.62, −0.47)	13.92 (5.61, 23.03)	11.62 (5.02, 18.99)	−0.60 (−0.68, −0.53)
Southern Latin America	14.68 (6.15, 24.47)	20.30 (8.95, 33.13)	1.40 (1.25, 1.54)	13.69 (5.60, 22.98)	19.36 (8.27, 31.70)	1.33 (1.22, 1.44)	15.71 (6.48, 26.86)	21.27 (9.41, 35.51)	1.45 (1.24, 1.66)
High-income North America	16.80 (7.19, 26.71)	23.06 (10.55, 35.25)	1.20 (1.10, 1.29)	14.64 (6.28, 22.99)	19.35 (8.79, 29.45)	1.10 (1.01, 1.20)	19.02 (8.09, 30.35)	26.85 (12.22, 41.24)	1.26 (1.16, 1.36)
Caribbean	10.54 (4.42, 17.00)	15.27 (6.49, 25.36)	1.34 (1.23, 1.44)	11.53 (4.83, 18.85)	16.40 (7.06, 27.60)	1.30 (1.19, 1.40)	9.50 (3.98, 15.47)	14.11 (5.85, 23.78)	1.39 (1.26, 1.52)
Andean Latin America	7.68 (3.08, 12.90)	12.20 (5.41, 20.38)	1.49 (1.34, 1.64)	8.44 (3.37, 14.72)	12.60 (5.39, 21.51)	1.20 (1.03, 1.37)	6.90 (2.58, 11.69)	11.78 (5.01, 20.64)	1.84 (1.69, 1.99)
Central Latin America	7.66 (3.19, 12.30)	17.46 (7.77, 27.76)	2.75 (2.64, 2.85)	8.16 (3.36, 12.97)	16.21 (7.31, 26.08)	2.33 (2.18, 2.49)	7.13 (2.94, 11.68)	18.81 (8.02, 29.83)	3.17 (3.08, 3.26)
Tropical Latin America	8.91 (3.60, 14.51)	17.55 (7.50, 27.64)	1.95 (1.82, 2.08)	9.33 (3.72, 15.36)	17.68 (7.51, 28.34)	1.77 (1.64, 1.90)	8.48 (3.32, 14.19)	17.41 (7.41, 27.73)	2.15 (1.99, 2.30)
North Africa and Middle East	10.97 (4.41, 18.52)	13.61 (5.74, 21.71)	0.81 (0.65, 0.97)	13.29 (5.32, 22.45)	14.65 (6.25, 23.20)	0.46 (0.29, 0.64)	8.78 (3.47, 14.97)	12.70 (5.28, 20.50)	1.25 (1.11, 1.40)
South Asia	1.59 (0.55, 2.64)	3.26 (1.24, 5.27)	2.32 (2.26, 2.38)	2.26 (0.76, 3.95)	3.96 (1.47, 6.69)	1.71 (1.58, 1.83)	1.00 (0.31, 1.78)	2.58 (0.96, 4.38)	3.23 (3.07, 3.39)
Central Sub-Saharan Africa	2.02 (0.71, 3.58)	4.48 (1.64, 7.99)	2.61 (2.43, 2.80)	2.09 (0.71, 3.81)	4.31 (1.54, 7.96)	2.35 (2.23, 2.47)	1.96 (0.61, 3.67)	4.64 (1.62, 8.95)	2.87 (2.61, 3.14)
Eastern Sub-Saharan Africa	3.16 (1.09, 5.34)	4.90 (1.80, 8.32)	1.19 (1.08, 1.29)	3.82 (1.37, 6.82)	5.12 (1.92, 8.80)	0.69 (0.56, 0.81)	2.48 (0.78, 4.33)	4.68 (1.70, 8.21)	1.87 (1.80, 1.94)
Southern Sub-Saharan Africa	10.32 (4.21, 16.33)	15.05 (6.13, 24.10)	1.69 (1.32, 2.06)	12.66 (5.22, 20.30)	17.43 (7.46, 28.63)	1.90 (1.35, 2.45)	7.83 (3.00, 12.99)	12.61 (4.99, 20.90)	1.45 (1.18, 1.71)
Western Sub-Saharan Africa	2.15 (0.84, 3.51)	3.77 (1.42, 6.38)	1.91 (1.85, 1.96)	2.81 (1.12, 4.66)	4.18 (1.58, 7.32)	1.32 (1.27, 1.38)	1.56 (0.55, 2.65)	3.34 (1.23, 5.75)	2.58 (2.50, 2.66)

ASDR, age-standardized DALY rate; EO-CRC, early-onset colorectal cancer; BMI, body mass index; SDI, socio-demographic index; GBD, Global Burden of Disease; EAPC, estimated annual percentage change.

A striking sex divergence emerged in the high-middle SDI group: female rates decreased slightly (EAPC: −0.52%, 95% CI: −0.67 to −0.36), while male rates continued to rise (EAPC: 1.09%, 95% CI: 0.95–1.23). Significant regional heterogeneity was evident. In 2021, the highest ASDRs were recorded in High-income North America (23.06 per 100,000), Southern Latin America (20.30 per 100,000), and Tropical Latin America (17.55 per 100,000), whereas South Asia (3.26 per 100,000) and Central, Eastern, and Western Sub-Saharan Africa (3.77–4.90 per 100,000) had the lowest rates. Central Latin America experienced the steepest increase over time (EAPC: 2.75%, 95% CI: 2.65–2.86), followed by Central Sub-Saharan Africa (EAPC: 2.61%, 95% CI: 2.43–2.80). In contrast, significant declines were observed in Central Asia (EAPC: −1.00%, 95% CI: −1.14 to −0.86) and Western Europe (EAPC: −0.58%, 95% CI: −0.66 to −0.49). Sex-specific trends amplified these patterns. Males exhibited a higher and faster-growing burden in every region. The most pronounced increase in males occurred in South Asia (EAPC: 3.23%, 95% CI: 3.07–3.39), whereas the peak female increase was observed in Central Sub-Saharan Africa (EAPC: 2.35%, 95% CI: 2.23–2.47).

**Figure 2:**
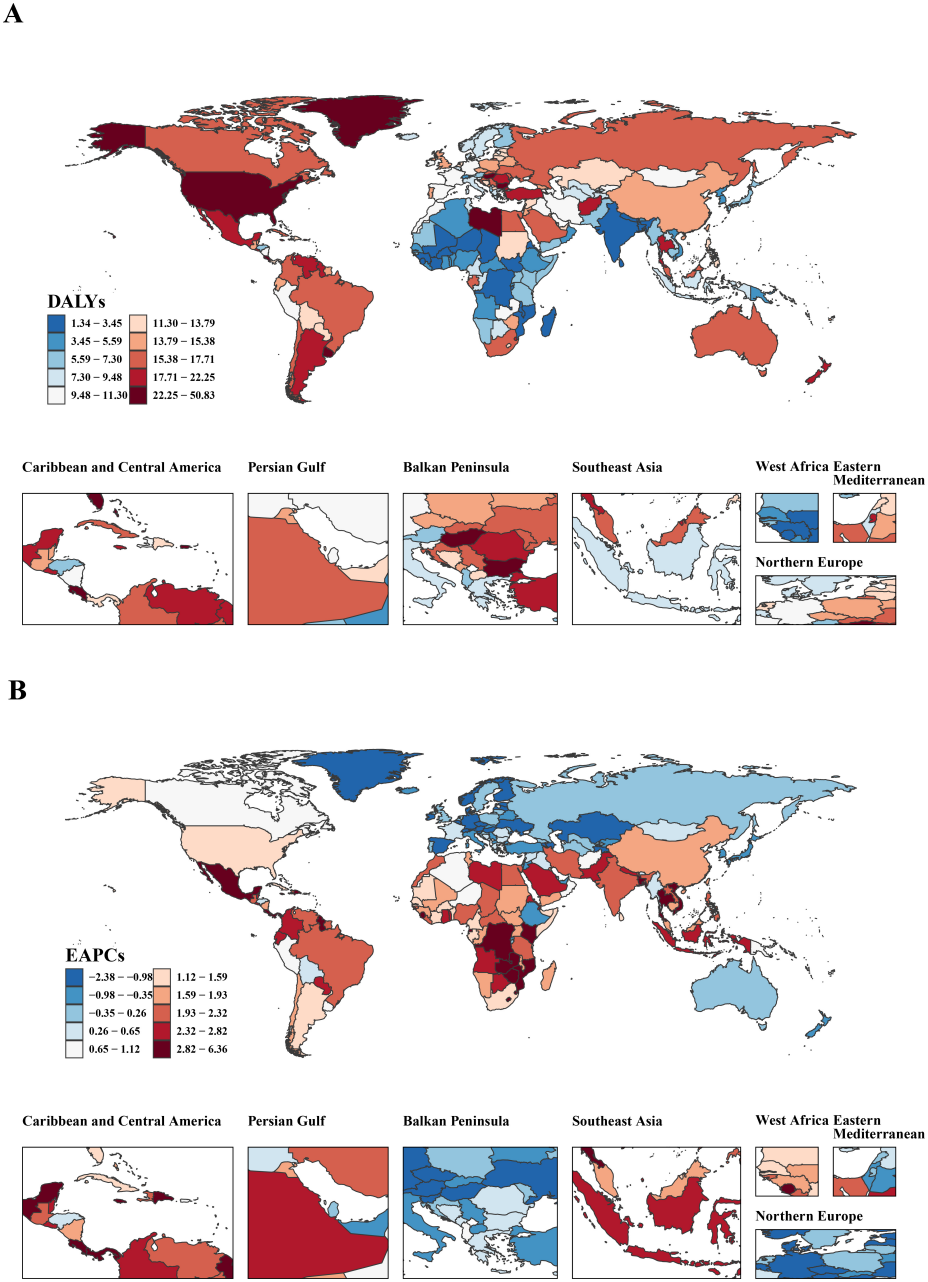
The burden of EO-CRC attributable to high BMI in 204 countries and territories. **(A)** The ASDRs in 204 countries and territories in 2021; **(B)** The EAPC in 204 countries and territories from 1990 to 2021. ASDR, age-standardized DALY rate; EAPC, estimated annual percentage change.

In 2021, the ASDRs for EO-CRC attributable to high BMI were analyzed across 204 countries (Figure 2A). Nauru had the highest DALY rate at 50.33 per 100,000 (95% UI: 18.72–91.29), followed by American Samoa (40.06 per 100,000, 95% UI: 18.09–67.95) and the Bahamas (32.88 per 100,000, 95% UI: 13.15–55.02). Conversely, countries like Burundi (2.04 per 100,000, 95% UI: 0.56–4.15), Niger (1.96 per 100,000, 95% UI: 0.65–4.01), and Mozambique (1.35 per 100,000, 95% UI: 0.49–2.63) had the lowest DALY rates, suggesting a lower impact of high BMI on EO-CRC in these regions. The EAPC showed notable differences in global trends (Figure 2B). Lesotho had the highest EAPC of 6.30 (95% CI: 5.51–7.11), followed by Zimbabwe (4.82, 95% CI: 3.76–5.89) and Vietnam (4.32, 95% CI: 4.00–4.65), all displaying significant increases in the EAPC values, indicating rising health risks from obesity in these regions. Meanwhile, countries like Czechia (−1.94, 95% CI: −2.45 to −1.44), Estonia (−1.95, 95% CI: −2.25 to −1.65), and Luxembourg (−2.36, 95% CI: −2.64 to −2.08) showed the lowest EAPC values.

We performed a correlation analysis between the SDI, DALYs, and EAPC across countries (Supplementary Figures S2A, B). In 2021, SDI and DALYs showed a significant positive correlation (*R* = 0.44, *P* < 0.001), suggesting a higher burden of high BMI-related EO-CRC in higher SDI countries (Supplementary Figure S2A). Between 1990 and 2021, SDI and EAPC exhibited a significant negative correlation (*R* = −0.43, *P* < 0.001), indicating that the rate of increase in high BMI-related EO-CRC may be slowing in high-SDI countries, while the disease burden in low-SDI countries warrants increased attention (Supplementary Figure S2B).

### 3.3 The burden of EO-CRC attributable to high BMI by different age and gender group

**Figure 3:**
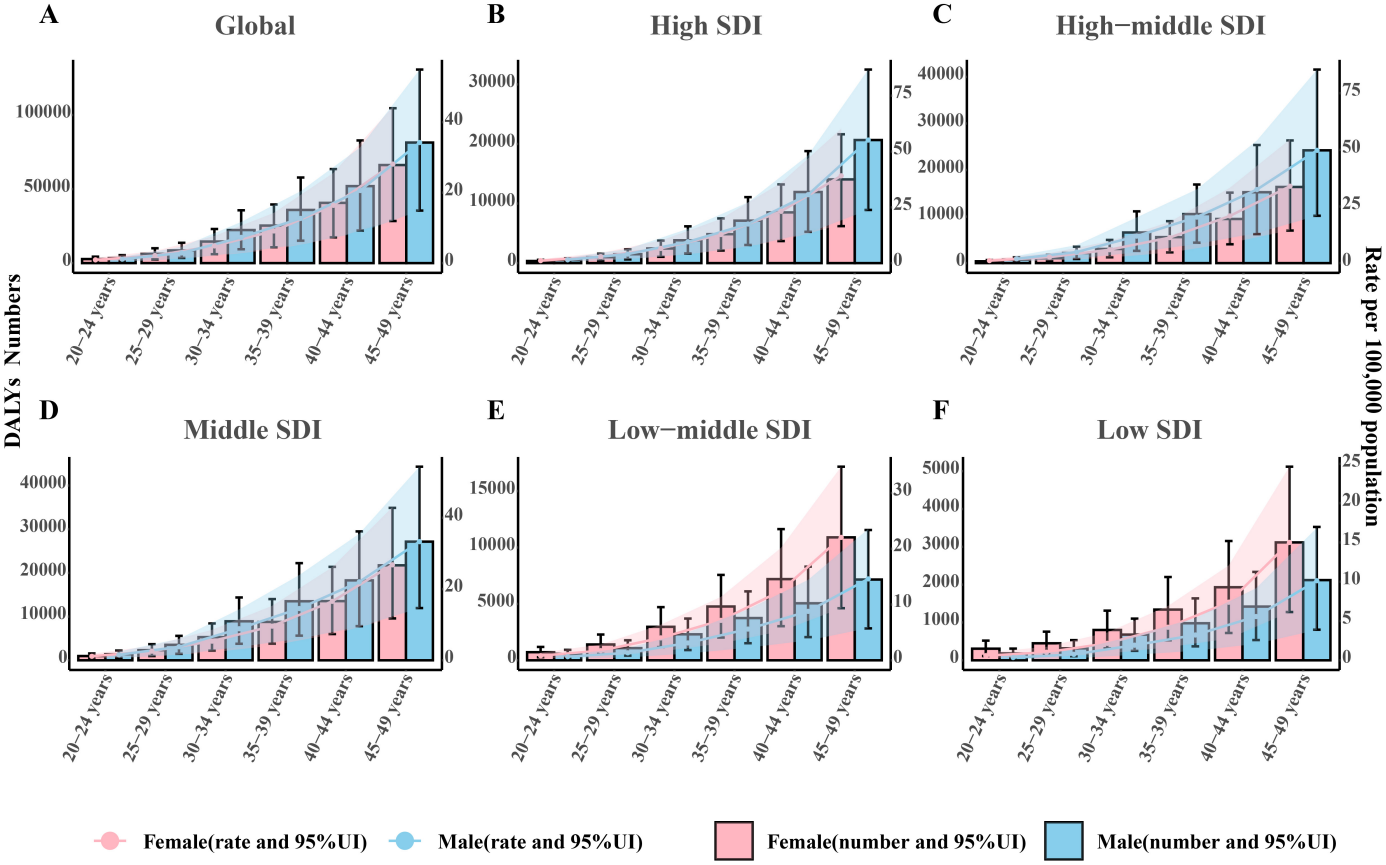
Age-specific DALY numbers and rates of early-onset colorectal cancer attributable to high body-mass index in **(A)** Global, **(B)** High SDI, **(C)** High-middle SDI, **(D)** Middle SDI, **(E)** Low-middle SDI, and **(F)** Low SDI regions. Bars represent sex-specific DALY numbers with 95% uncertainty intervals (UIs): pink for females, blue for males. Superimposed solid lines depict the corresponding DALY rates per 100,000 populations (right y-axis) with shaded 95% UIs (pink, females; blue, males). Age groups are 20–24, 25–29, 30–34, 35–39, 40–44, and 45–49 years. UI, uncertainty interval.

The burden of EO-CRC attributable to high BMI increased monotonically with age in 2021 across the global and five SDI regions (Figures 3A–F). Globally, total DALYs rose from 6,149.46 in the 20–24 years age group to 147,635.43 in the 45–49 years group, with the rate curve climbing from 1.03 to 31.18 per 100,000 (Figure 3A). A consistent male predominance was observed in the global, high, high-middle, and middle SDI regions. The male-to-female rate ratio reached 1.41 in the high SDI region (55.12 vs. 39.11 per 100,000) and 1.47 in the high-middle SDI region (49.78 vs. 33.93 per 100,000) in the 45–49 years group. Conversely, this pattern reversed in less-developed settings. In the low-middle SDI region, female DALY rates exceeded male rates in every age band. The male-to-female rate ratio was 0.66 (14.59 vs. 22.04 per 100,000) in the 45–49 years group in this region. A similar pattern was observed in the low SDI region, where the male-to-female rate ratio was 0.68 (10.13 vs. 15.00 per 100,000). These contrasting sex profiles indicate that the well-established male excess of high BMI mediated CRC in affluent populations is not universal; in lower-resource settings, young women are increasingly affected. This highlights the need for sex-specific obesity and cancer control strategies in these regions.

We further explored the proportion of DALY numbers attributable to high BMI for EO-CRC at different age groups in 1990 and 2021 across 21 GBD regions. The results suggest that, globally, and in 18 of the 21 GBD regions analyzed, with the exception of Tropical Latin America, the Caribbean, and Southern Sub-Saharan Africa, the DALY numbers in the 40–49 years age group have declined (Supplementary Figure S3). This trend points to a younger age profile for the burden.

### 3.4 Joinpoint analysis

**Figure 4:**
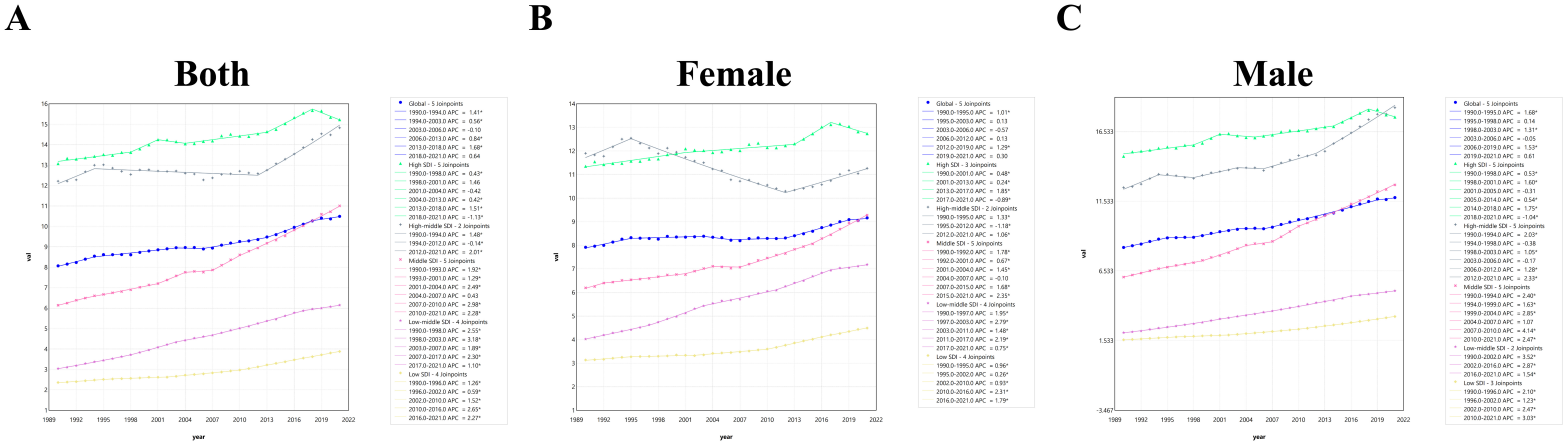
Joinpoint regression of ASDRs for EO-CRC attributable to high BMI, 1990–2021, shown for **(A)** both sex, **(B)** male, and **(C)** female at the global level and across five SDI regions. APC, annual percent change; AAPC, average annual percent change.

Joinpoint regression of ASDRs for EO-CRC attributable to high BMI revealed a sustained global increase from 1990 to 2021 (Figure 4A, Supplementary material). Over the entire period the average annual percent change (AAPC) was 0.86% (95% CI: 0.68–1.03, *P* < 0.001), but the magnitude of growth escalated step-wise with lower socio-demographic development: AAPC rose from 0.46% in high-SDI regions to 0.69% in high-middle SDI, 1.90% in middle SDI, 2.30% in low-middle SDI and 1.63% in low SDI. Consistent with this gradient, the steepest period-specific increases occurred in middle SDI between 2007 and 2010 (APC: 2.98%) and in low-middle SDI between 1998 and 2003 (APC: 3.18%).

Sex-stratified analyses showed markedly faster growth among males (AAPC: 1.17 % vs. 0.47 %) than females (AAPC: 0.47%) in global analysis (Figures 4B, C, Supplementary material). Male rates climbed most rapidly in middle SDI (AAPC: 2.41%) and low-middle SDI (AAPC: 2.91%); the largest single-segment surge was 4.14% during 2007–2010 in middle SDI, followed by 3.03% during 2010–2021 in low SDI (Figure 4C). Female increases were smaller but still pronounced in middle (AAPC: 1.33%) and low-middle SDI (AAPC: 1.88%); high-middle SDI females showed no net change (AAPC: −0.13%, *P* = 0.09). Notably, high-SDI trajectories reversed after 2017–2018, with significant recent declines in both sexes (APC: −1.13% in females and −1.04% in males).

Taken together, the Joinpoint analysis indicates that the DALY burden of EO-CRC attributable to high BMI is rising worldwide, driven predominantly by rapid gains in middle- and low-middle SDI settings and by consistently higher growth rates in males, underscoring widening disparities across both socio-economic and sex strata.

### 3.5 Decomposition analysis

**Figure 5:**
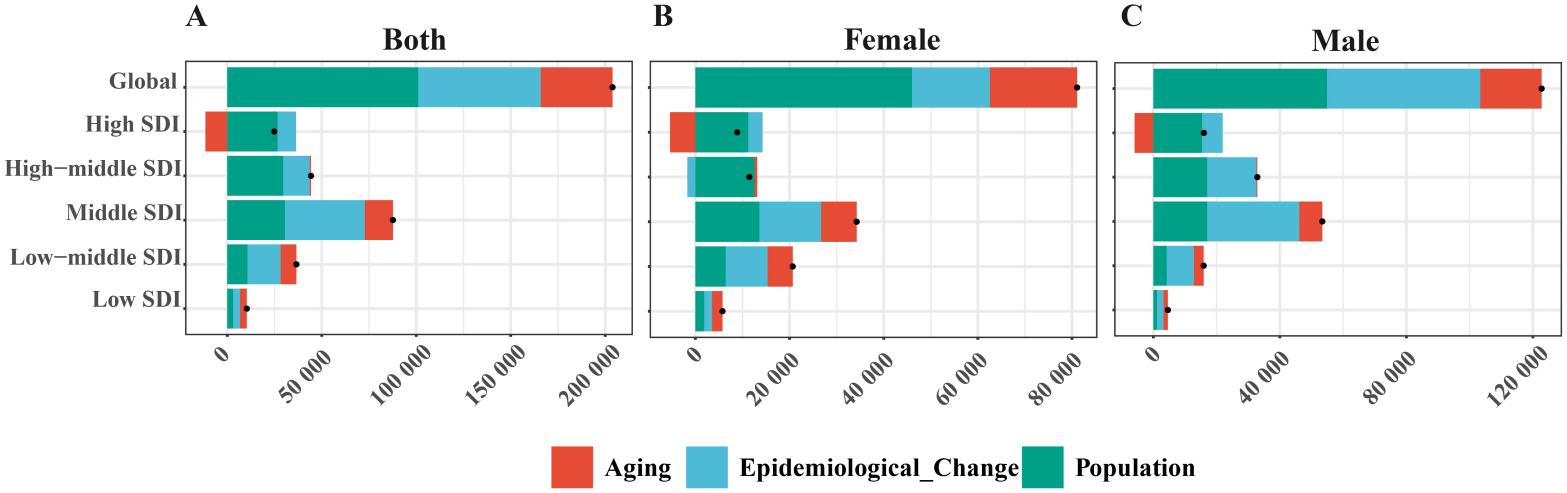
Decomposition analysis of population growth (green), epidemiological change (blue), and population aging (red) to the absolute change in DALYs for EO-CRC attributable to high BMI, 1990–2021. Bars display **(A)** both sex, **(B)** female, and **(C)** male results for the global and five SDI regions; values left of zero indicate a reduction. Black dots mark net change for each stratum.

From 1990 to 2021, the number of DALYs attributable to high BMI in EO-CRC rose by 203,873.31 globally: 49.56% from population growth, 31.78% from epidemiological change and 18.67% from aging (Figure 5A). In the high SDI regions, aging was negative (−46.52%), population growth (107.53%), and epidemiological change (38.99%) drove a net gain of 24,835. In the high-middle SDI regions, population growth was dominated factor (66.83%) and aging was neutral. In the middle and low-middle SDI regions, epidemiological change became the single largest driver (48.04% and 47.79%, respectively). In the low SDI regions, all three factors contributed comparably. Sex-stratified analysis shows a larger absolute rise in males (122,779.39) than females (81,093.92, Figures 5B, C). In males, epidemiological change (39.41%) nearly matched population growth (44.76%) worldwide and surpassed it in middle-income settings; in females, growth was the dominant driver globally (56.78%), and epidemiological change was negative in high-middle SDI regions (−14.98%). Negative aging effects remained confined to high SDI regions in both sexes.

For the analysis of 21 GBD regions, East Asia contributed the largest increase (63,617.54), primarily due to epidemiological change (63.34%), while aging had a slight negative effect (−4.16%) (Supplementary Figure S4A). North Africa and Middle East (27,413.03) and Southeast Asia (23,126.50) followed, driven mainly by aging (48.03%) and epidemiological change (48.56%), respectively. In high-income North America and Western Europe, population growth dominated, while aging contributed negatively (−40.81% and −33.11%). Central Asia showed minimal net change (1,301.11) due to negative epidemiological improvements. In males, epidemiological change was the leading driver in several GBD regions, including East Asia (70.30%) and Southeast Asia (Supplementary Figure S4C, 53.03%). By contrast, in females, population growth was the predominant driver across nearly all regions (56.78%), with aging contributing more substantially than in males (Supplementary Figures S4B, C, 22.83% vs. 15.83%).

### 3.6 Frontier analysis

**Figure 6:**
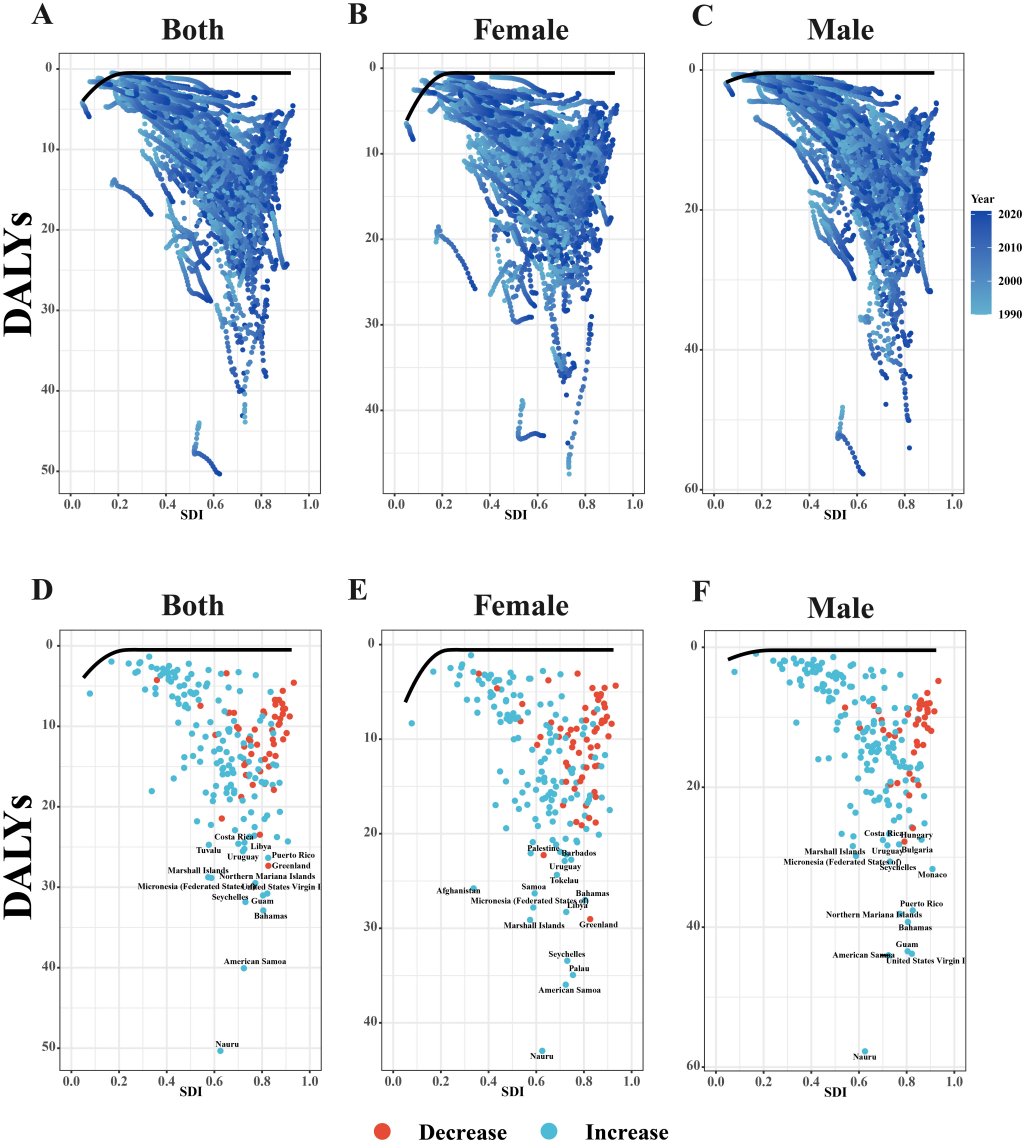
Frontier analysis of ASDRs attributable to high BMI across countries from 1990 to 2021 for **(A)** both sex, **(B)** female, and **(C)** male. The changes in ASDRs from 1990 to 2021 for **(D)** both sex, **(E)** female, and **(F)** male, with countries showing an decrease in ASDRs marked in blue and those with a increase in red.

Across 1990–2021, national ASDRs for EO-CRC attributable to high BMI rose as the SDI rose (Figure 6A). Sex-stratified analyses reveal steeper improvements in females: country–year paths cluster tightly around the frontier at SDI ≥ 0.6, whereas male paths remain more dispersed and consistently above female values (Figures 6B, C).

Net-change panels confirm this disparity (Figures 6D–F). A larger share of countries achieved DALY reductions in females, while male increases were concentrated in Oceania and parts of Central America (Figures 6E, F). The greatest declines (red points) for both sexes were seen in higher-income Caribbean settings (e.g., Barbados, Curaçao), whereas the largest male rises occurred in Samoa and Nauru. Thus, although rising SDI generally coincides with falling DALY burden, progress is uneven, with males and selected island nations lagging behind the global frontier.

### 3.7 BAPC analysis

**Figure 7:**
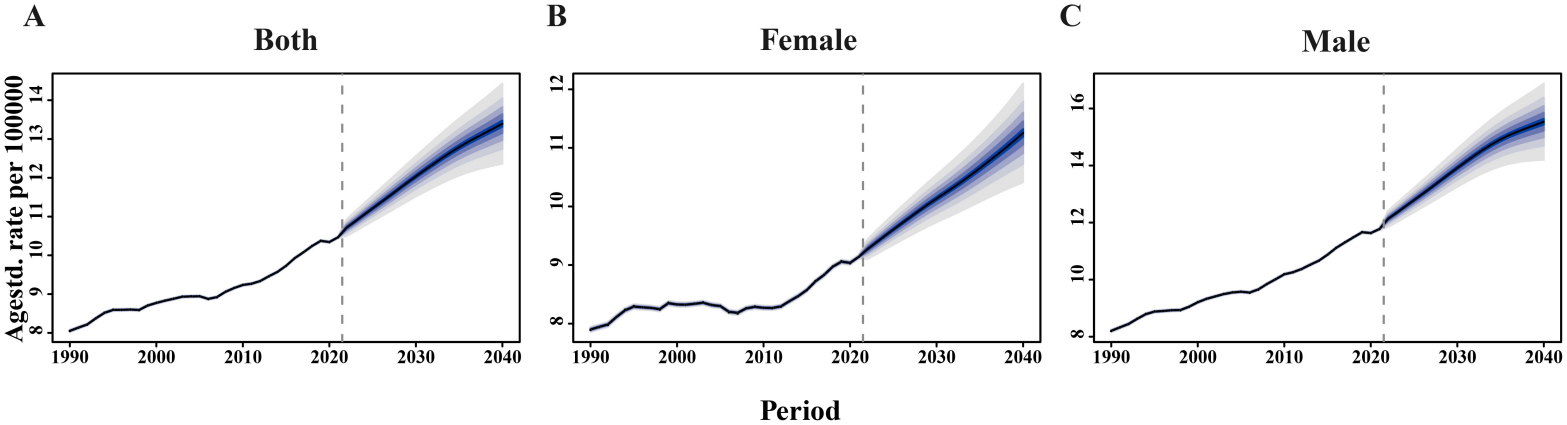
Bayesian Age-Period-Cohort (BAPC) projections of ASDRs for EO-CRC attributable to high BMI for **(A)** both sex, **(B)** female, and **(C)** male from 1990 to 2040. The shaded regions represent the uncertainty interval. The dashed vertical line indicates the year 2021.

BAPC analysis was performed to predict the ASDRs for EO-CRC attributable to high BMI by 2040. These projections underscore the increasing future burden of EO-CRC attributable to high BMI, particularly among males, with a rapid rise expected in the next two decades (Figures 7A–C). In both high- and middle-SDI regions, the burden showed a declining trend for both males and females. By contrast, in low- and low-middle SDI regions, an upward trend was observed in both sexes. Notably, in high and middle-high SDI regions, gender-specific differences were apparent: the trend remained relatively stable in males but declined more significantly in females (Supplementary Figure S5).

## 4 Discussion

This global assessment reveals that the burden of EO-CRC attributable to high BMI has risen substantially over the past three decades, with notable geographic and demographic disparities. Between 1990 and 2021, the ASDRs increased by 30% globally, reflecting the worldwide surge in young adult CRC incidence that has been documented in numerous countries. High BMI emerged as the leading modifiable risk factor driving the increase in EO-CRC burden in 24 of 27 global regions analyzed, surpassing other lifestyle risks. Our findings underscore that the global obesity epidemic has become a key contributor to the rise of CRC in adults under 50. We observed that high-income countries currently exhibit the highest absolute EO-CRC burden linked to obesity, yet their growth in rates has begun to plateau or even reverse in recent years. In contrast, lower SDI regions experienced the most rapid increases in BMI-attributable EO-CRC (EAPC 2.30% in low-middle SDI vs. 0.53% in high SDI), signaling a widening disparity as the obesity and cancer transition shifts to low- and middle-income countries. Notably, a consistent global male predominance in the burden of EO-CRC was observed, with men exhibiting higher DALY rates and a more rapid increase over time compared to women. However, this male predominance was not universal: in low SDI regions, young women now experience comparable or even higher obesity-related EO-CRC rates than men.

Previous large-scale studies have either examined CRC trends in the overall population or focused on early-onset disease without in-depth risk factor attribution (3, 16–23). Junhai et al. focused on the very early onset CRC and found that high BMI was one of the main contributors to deaths and DALYs (24). Regional studies have reported a marked increase in EO-CRC, with particularly poor outcomes among young females in Southeast Asia (25). This trend aligns, to some extent, with our findings. Specifically, in Southeast Asia, the DALYs of EO-CRC attributable to high BMI are higher in females than in males. A study examining the risk factors contributing to the burden of EO-CRC in Gulf Cooperation Council countries also highlighted the significant role of high BMI among males (26). A meta-analysis provided robust evidence linking BMI with EO-CRC, showing a notably higher risk for obesity (OR: 1.88, 95% CI: 1.40–2.54) than for overweight (OR: 1.32, 95% CI: 1.19–1.47) (27). Unlike previous studies, which were often limited to specific regions or populations, our study draws on data from 204 countries to provide a comprehensive global analysis of EO-CRC, highlighting the substantial burden attributable to high BMI. It provides nuanced insights (by age, gender, region, and time) to inform targeted interventions.

The strong association between excess adiposity and colorectal carcinogenesis observed in our study is biologically plausible and supported by mechanistic research. Obesity leads to a cascade of metabolic dysregulation that can promote tumor development in the colon and rectum (28). One key pathway is insulin resistance and hyperinsulinemia: adiposity, especially visceral fat, drives chronic insulin resistance, leading to elevated circulating insulin and IGF-1 levels, which have mitogenic and anti-apoptotic effects on colonic epithelial cells (29). Obesity also creates a state of chronic low-grade inflammation-excess adipose tissue secretes pro-inflammatory cytokines (e.g., IL-6, TNF-α) and alters adipokine profiles (leptin up, adiponectin down), fostering a microenvironment conducive to neoplastic growth (30). These mechanistic links help explain why the rise of obesity parallels an upswing in EO-CRC incidence. The changing lifestyles of the past few decades-characterized by greater consumption of ultra-processed, energy-dense foods, and more sedentary routines-have led to higher BMI at younger ages globally. Consequently, younger generations are experiencing prolonged exposure to obesogenic metabolic and inflammatory conditions that can initiate colorectal neoplasia earlier in life.

Sex differences in obesity-related carcinogenesis warrant special consideration. Biological distinctions between men and women may modulate how excess weight translates into cancer risk. Men tend to accumulate visceral fat more readily, whereas premenopausal women carry proportionally more subcutaneous fat (31). Visceral adiposity is metabolically more active and pro-inflammatory; it correlates strongly with insulin resistance and colorectal tumorigenesis (32). This could partly explain why historically and globally, males have higher CRC incidence than females. On the other hand, female sex hormones and other physiological differences may offer some protection in early adulthood-for example, estrogen has been hypothesized to exert anti-inflammatory and insulin-sensitizing effects in the colon, potentially delaying carcinogenesis in women (33). However, as obesity prevalence in women increases (and as women age into menopause, losing the hormonal advantage), their CRC risk may rise rapidly. Notably, one cohort study reported that women with obesity (BMI ≥30) had nearly double the risk of EO-CRC compared to normal weight women, illustrating that excess weight confers significant risk in females as well (34). Furthermore, disparities in healthcare access and cancer awareness between sexes can influence outcomes. In certain low-resource settings, young women may face greater barriers to timely diagnosis, which might contribute to the higher female EO-CRC burden we observed in low SDI regions. The interplay of adiposity, hormones, fat distribution, and healthcare factors in CRC carcinogenesis is complex, and further research is needed to clarify these sex-specific mechanisms. What is clear is that obesity's carcinogenic effects do not spare either sex—reinforcing the need for weight control in both young men and women.

Our study revealed pronounced geographic heterogeneity in EO-CRC trends attributable to high BMI. High-income countries still exhibit the highest ASDR today, reflecting historically elevated baseline risks. However, the growth of EO-CRC in many of these high SDI regions has begun to stagnate or slow relative to lower SDI regions. This phenomenon may be attributed to widespread screening efforts and increased public awareness, which have likely contributed to earlier detection and reporting. Since approximately 2017, many high-SDI countries have implemented public anti-obesity interventions aimed at addressing rising obesity rates. For instance, the United Kingdom introduced a sugar-sweetened beverage (SSB) tax aimed at reducing sugary drink consumption. Chile enacted the Law of Food Labeling and Advertising, which not only established the first mandatory front-of-package warning label system but also imposed strict marketing restrictions and banned the sale and promotion of high-sugar, high-sodium, high-fat, or high-calorie products in schools. In the United States, the “Smart Snacks” policy was adopted to limit access to unhealthy foods in the school environment. In contrast, low and middle SDI countries are now witnessing the steepest increases in EO-CRC burden as they pass through the later stages of the “epidemiologic transition.” Rapid economic development in parts of Asia, Latin America, the Middle East, and sub-Saharan Africa has brought about swift shifts in nutrition and lifestyle, often outpacing public health preparedness. The dietary shift from traditional high-fiber diets to Westernized patterns rich in red/processed meats and ultra-processed foods in these regions is closely associated with rising rates of obesity and CRC. Similarly, regions like South Asia and parts of sub-Saharan Africa, which historically had very low CRC incidence, are experiencing a concerning upswing in EO-CRC cases as obesity and other lifestyle risks proliferate. This suggests that without intervention, the gap in CRC burden between high- and low-SDI regions will continue to narrow in coming years, potentially even reversing, as the developing world faces a growing wave of EO-CRC. Our findings reinforce that EO-CRC is truly a globalizing problem—one that is shifting from being a predominantly high-income issue to a new threat across low- and middle-income populations as lifestyles change.

Several limitations of this study should be considered when interpreting the findings. First, the study relied on GBD's modeled estimates for high BMI-attributable EO-CRC burden, which carry inherent uncertainty. In many low SDI countries, cancer registry data are sparse and risk factor exposures are not systematically surveyed, necessitating statistical modeling and imputation. Second, Diet and other metabolic risks often cluster with obesity, so some of the BMI-attributable burden may overlap with or be mediated by these co-factors. The results of this study represent the estimation of the burden independently attributable to high BMI within the framework of the GBD risk attribution methodology, rather than the absolute causal effect after excluding all potential influences of lifestyle factors. Third, the frontier analysis, while novel, has its own limitations in attributing efficiency scores to countries-differences in data quality or unaccounted contextual factors could influence those results. Fourth, our study focuses solely on the disease burden attributable to high BMI, rather than the absolute burden difference between high and normal BMI populations. The conclusions of this study reflect the attribution of EOCRC burden to high BMI as estimated under the GBD risk attribution framework and should not be interpreted as a direct comparison between high and normal BMI groups.

Based on our findings, we recommend the development of sex-specific strategies for the prevention of obesity and CRC, the reinforcement of existing obesity control measures in high-income countries alongside the early implementation of preventive efforts in low- and middle-income countries, greater emphasis on obesity prevention among children and adolescents, and the consideration of initiating CRC screening earlier in high-risk populations.

In conclusion, this study assessed the global burden of EO-CRC attributable to high BMI. The results indicate that high BMI is a major modifiable risk factor contributing to the increasing burden of EO-CRC, with marked variations by gender and region. Although the burden is generally higher in males than in females, distinct patterns emerge across different SDI regions. These findings underscore the critical role of high BMI in EO-CRC prevention and highlight the necessity of implementing targeted intervention strategies tailored to specific sexes and regions.

## Data Availability

The original contributions presented in the study are included in the article/Supplementary material, further inquiries can be directed to the corresponding author.

## Supplementary material

Supplementary Figure S1 Ranked contribution of DALY variation in EO-CRC by 11 risk factors (1990–2021) for **(A)** females and **(B)** males.

Supplementary Figure S2 Correlation analysis between the SDI and ASDRs in 2021, and between SDI and EAPC from 1990 to 2021 across countries and territories.

Supplementary Figure S3 The proportion of DALY numbers attributable to high BMI for EO-CRC at different age groups in 1990 and 2021 across 21 GBD regions.

Supplementary Figure S4 Decomposition of the absolute change in DALYs for early-onset colorectal cancer attributable to high BMI, 1990–2021, by population growth, epidemiological change, and aging for **(A)** both sexes, **(B)** females, and **(C)** males across 21 GBD regions. Bars extending left indicate negative contributions. Black dots represent net changes.

Supplementary Figure S5 BAPC projections of ASDRs for EO-CRC attributable to high BMI for **(A)** both sex, **(B)** female, and **(C)** male in high SDI regions; **(D)** both sex, **(E)** female, and **(F)** male in high-middle SDI regions; **(G)** both sex, **(H)** female, and **(I)** male in middle SDI regions; **(J)** both sex, **(K)** female, and **(L)** male in low-middle SDI regions; **(M)** both sex, **(N)** female, and **(O)** male in low SDI regions from 1990 to 2040. The shaded regions represent the uncertainty interval. The dashed vertical line indicates the year 2021.
