# Supplementary figures and images for: Global, regional, and national burden of early-onset colorectal cancer attributable to high BMI (1990–2021)
Source: Front Med (Lausanne). 2025 Sep 17;12:1631392. doi: 10.3389/fmed.2025.1631392 (PMC12484016; doi:10.3389/fmed.2025.1631392)

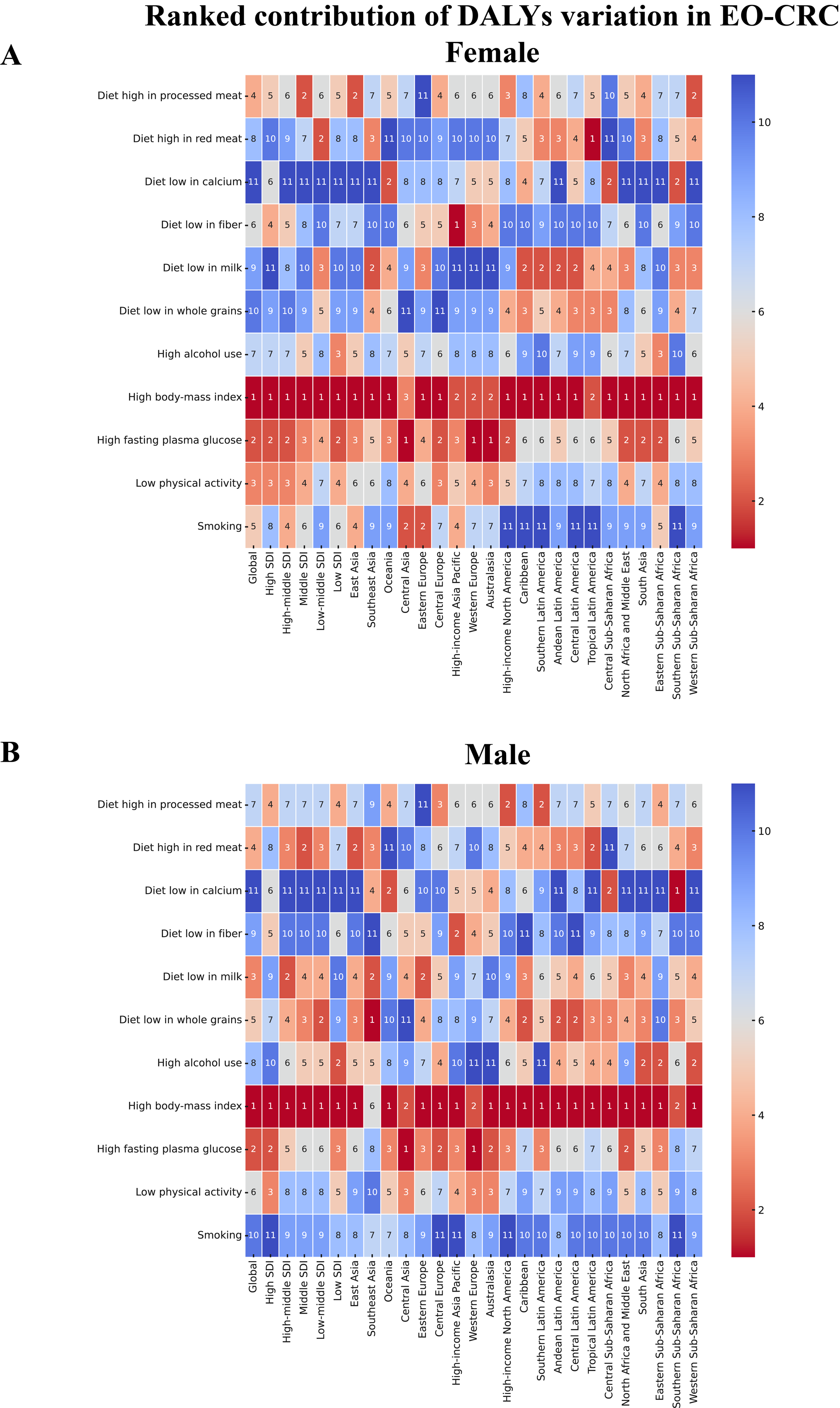

Supplement: Supplementary Figure S1 — Ranked contribution of DALY variation in EO-CRC by 11 risk factors (1990–2021) for (A) females and (B) males. [file Image_1.tif]

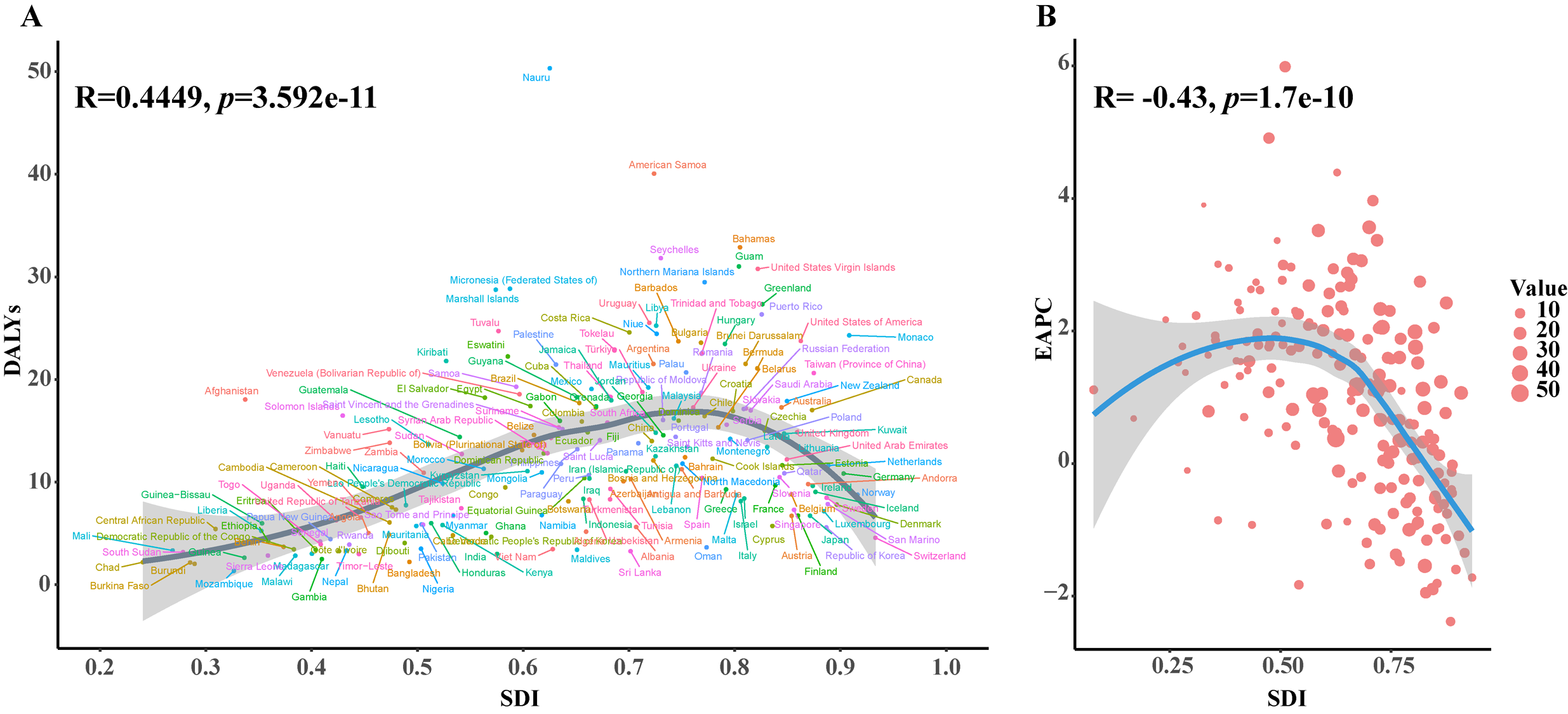

Supplement: Supplementary Figure S2 — Correlation analysis between the SDI and ASDRs in 2021, and between SDI and EAPC from 1990 to 2021 across countries and territories. [file Image_2.tif]

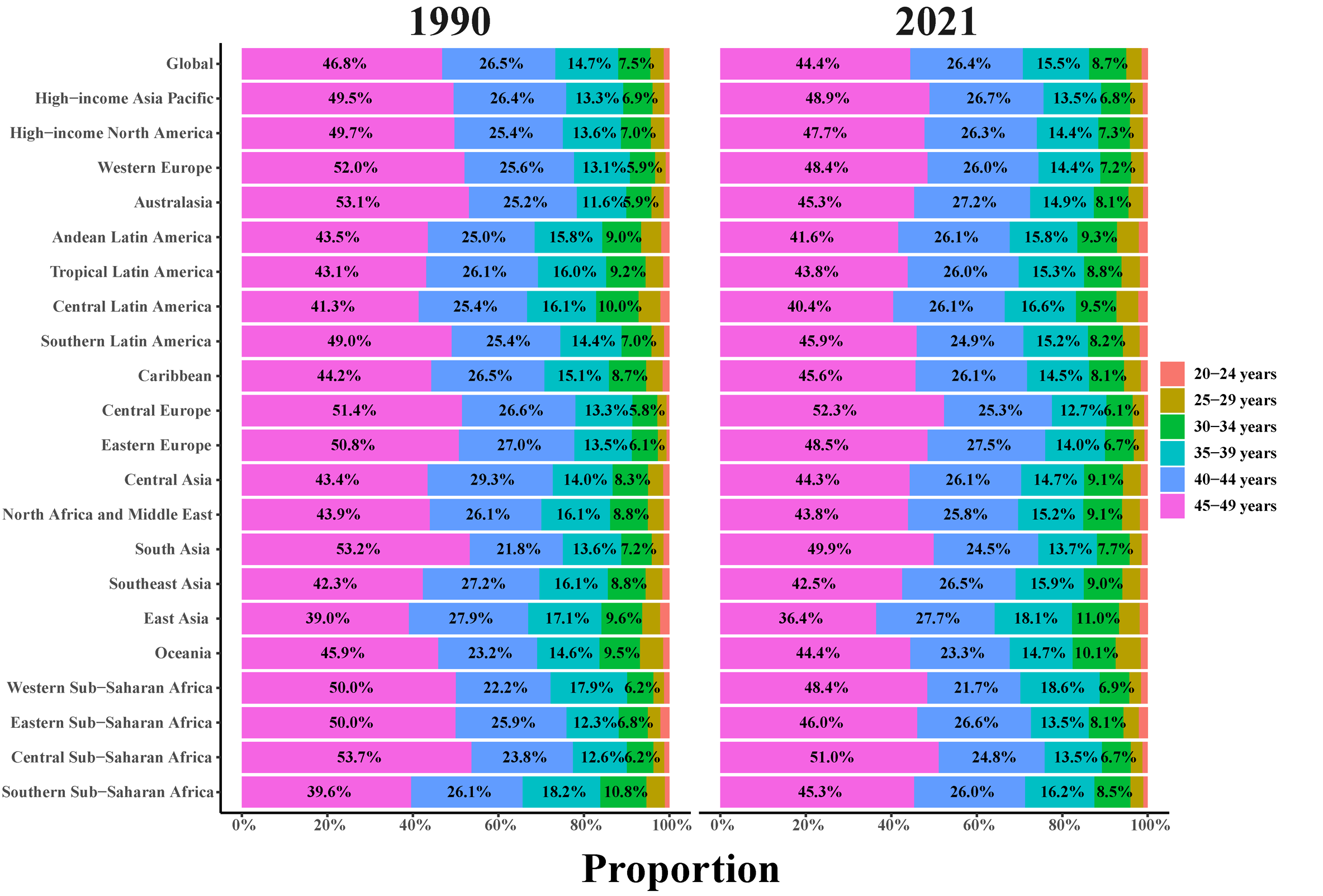

Supplement: Supplementary Figure S3 — The proportion of DALY numbers attributable to high BMI for EO-CRC at different age groups in 1990 and 2021 across 21 GBD regions. [file Image_3.tif]

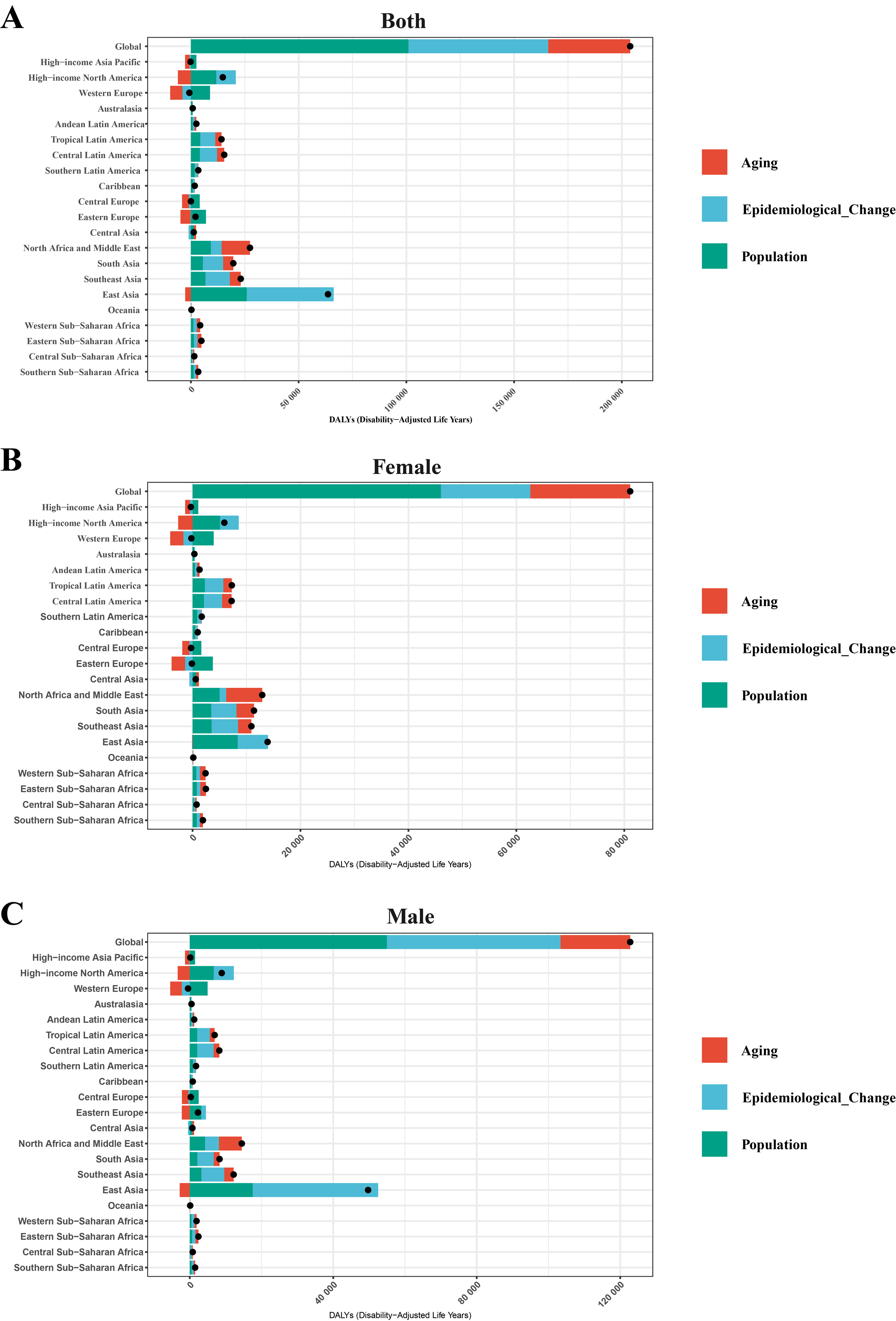

Supplement: Supplementary Figure S4 — Decomposition of the absolute change in DALYs for early-onset colorectal cancer attributable to high BMI, 1990–2021, by population growth, epidemiological change, and aging for (A) both sexes, (B) females, and (C) males across 21 GBD regions. Bars extending left indicate negative contributions. Black dots represent net changes. [file Image_4.tif]

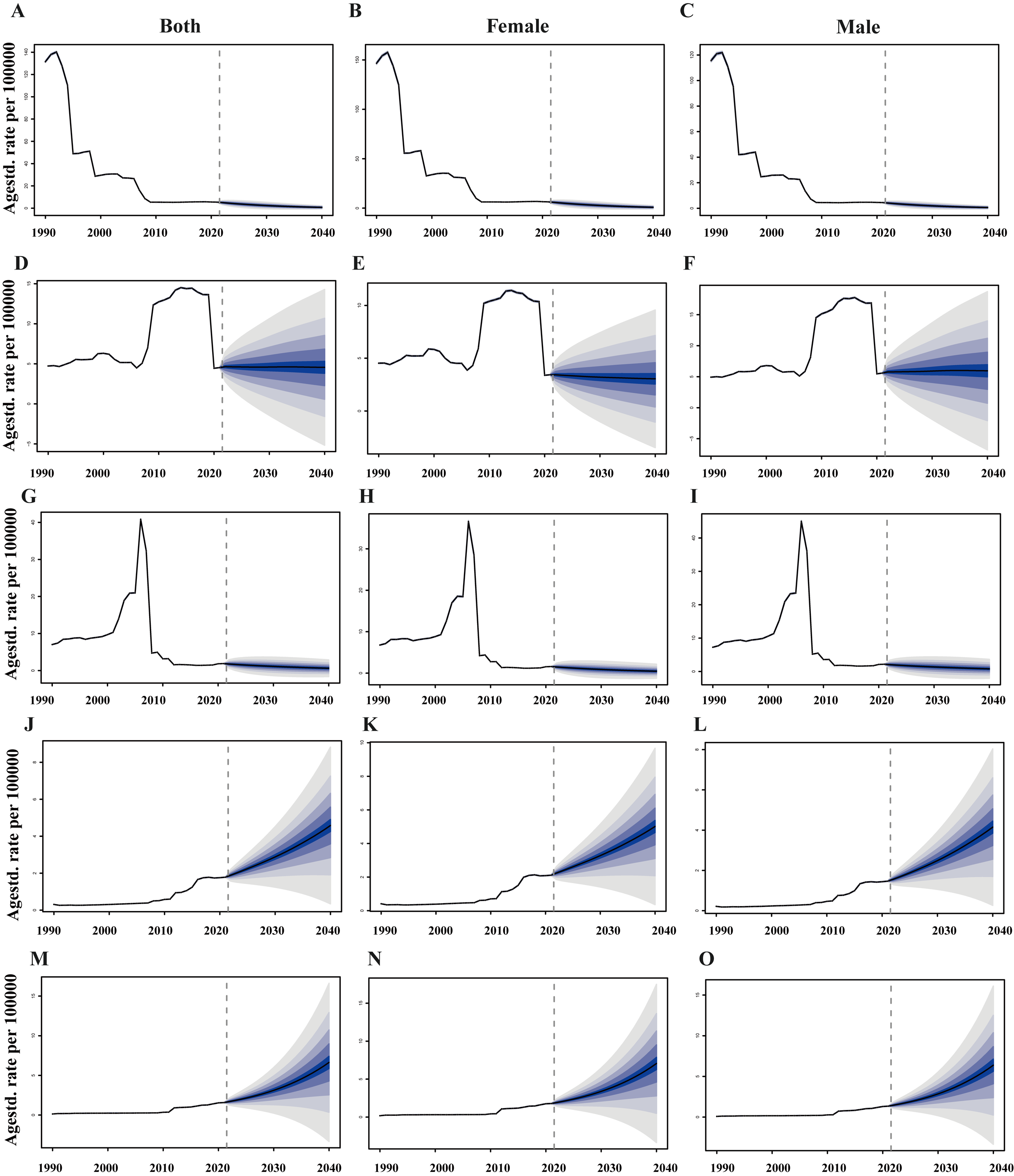

Supplement: Supplementary Figure S5 — BAPC projections of ASDRs for EO-CRC attributable to high BMI for (A) both sex, (B) female, and (C) male in high SDI regions; (D) both sex, (E) female, and (F) male in high-middle SDI regions; (G) both sex, (H) female, and (I) male in middle SDI regions; (J) both sex, (K) female, and (L) male in low-middle SDI regions; (M) both sex, (N) female, and (O) male in low SDI regions from 1990 to 2040. The shaded regions represent the uncertainty interval. The dashed vertical line indicates the year 2021. [file Image_5.tif]
